# Gastric Hyperplastic Polyp Associated with Proliferation of Xanthoma Cells Observed by Magnification Narrow-Band Imaging Endoscopy

**DOI:** 10.1155/2009/845260

**Published:** 2009-12-15

**Authors:** Shoji Hirasaki, Motoharu Kubo, Atsushi Inoue

**Affiliations:** Division of Gastroenterology, Kubo Hospital, 1-1-19 Uchibori, Imabari 799-2116, Japan

## Abstract

A case of gastric hyperplastic polyp with proliferation of xanthoma cells is reported. The patient was a 69-year-old man who visited our hospital for further evaluation of gastric polyps. Endoscopic examination of the upper digestive tract revealed multiple hyperplastic polyps in the gastric antrum. There was a pedunculated polyp with whitish yellow granules, 7 mm in diameter, arising from the greater curvature of the antrum. Magnification narrow-band imaging endoscopy (GIF-H260Z, Olympus) revealed long microcapillaries in the polyp but did not reveal disappearance of the mucosal microstructure or irregular branched capillaries. Endoscopic mucosal resection (EMR) was performed. Histological examination of the specimen revealed the lengthened gastric foveolae in the superficial portion and tight sheet of foamy histiocytes in the lamina propria. Diagnosis of gastric hyperplastic polyp with proliferation of xanthoma cells was made. There was no evidence of malignancy. It is necessary to know that a gastric hyperplastic polyp may associate with gastric xanthoma, although such association is very rare.

## 1. Introduction

Hyperplastic polyps constitute the most frequent type of gastric epithelial polyp [1–3]. These polyps are often multiple. To the best of our knowledge, gastric hyperplastic polyps are rarely associated with xanthoma cells [1, 4]. Herein, we report a rare case of a Japanese man diagnosed with gastric hyperplastic polyp with proliferation of xanthoma cells that observed by magnifying endoscopy with narrow-band imaging and treated with endoscopic mucosal resection (EMR). 

## 2. Case Report

**Figure 1:**
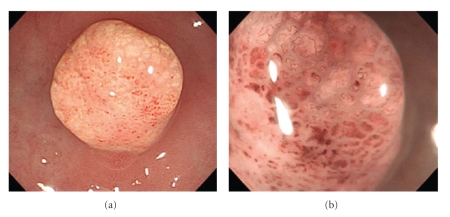
Endoscopic appearance of the pedunculated gastric hyperplastic polyp (7 mm in size) arising from the greater curvature of the antrum. (a) The polyp had whitish yellow granules. (b) Magnification narrow-band imaging endoscopy revealed long microcapillaries in the polyp but did not reveal disappearance of the mucosal microstructure or irregular branched capillaries (×50).

**Figure 2:**
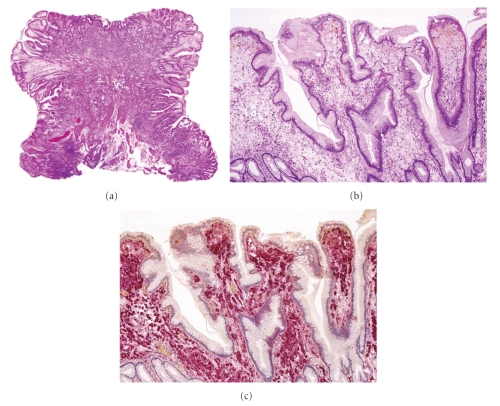
(a) The resected specimen obtained by endscopic mucosal resection showed lengthened gastric foveolae in the superficial portion in the polyp (HE stain, ×4). (b) Collections of large foamy histiocytes were seen in the lamina propria (HE stain, ×25). (c) The histiocytes represented a positive immunochemical reaction for CD68 (×25).

A 69-year-old man visited our hospital for the further evaluation of gastric polyps. His body temperature was 36.2°C, blood pressure was 126/72 mmHg, and radial pulse rate was 60 beats/min and regular. He had neither anemia nor jaundice. Neurological examination revealed no abnormal findings and there was no lymphadenopathy. No specific family history was identified. Routine hematological examination and biochemical tests were within normal limits. Serum anti-H. pylori immunoglobulin G (IgG) antibody was positive. Endoscopic examination of the upper digestive tract revealed multiple gastric hyperplastic polyps, 7–15 mm in size, in the gastric antrum. There was a pedunculated polyp with whitish yellow granules, 7 mm in diameter, arising from the greater curvature of the antrum (Figure 1(a)). The surface of the polyp was smooth and without irregular area. The color of the polyp was almost uniform, but red spots were seen in several places in the polyp. Magnification narrow-band imaging endoscopy (GIF-H260Z, Olympus) revealed long microcapillaries in the polyp but did not reveal disappearance of the mucosal microstructure or irregular branched capillaries (Figure 1(b)). We speculated that the benign polyp was associated with xanthoma cells. The patient underwent EMR. The protruding lesion, 7 × 6 mm in size, was resected completely with a safe lateral and vertical margin. Histological examination of the EMR specimen revealed lengthened gastric foveolae in the superficial portion (Figure 2(a)). Tight sheet of foamy histiocytes was seen in the superficial lamina propria (Figure 2(b)). Immunohistological studies were carried out for the resected specimen and the histiocytes represented a positive immunochemical reaction for CD68 (Figure 2(c)). The pedunculated polyp was diagnosed as a gastric hyperplastic polyp with xanthoma. There was no evidence of malignancy. The post-EMR course was uneventful. 

## 3. Discussion

Gastrointestinal xanthomas occur most frequently in the stomach, and rarely in the esophagus. Details of its developmental mechanism remain unknown. Gastric xanthoma may occur in any part of the stomach as rounded or oval, yellow or yellow-white macules or nodules measuring 1 to 5 mm in diameter [5]. Xanthomas are composed of foamy cells, termed xanthoma cells, characterized by abundant vacuolated cytoplasm. The xanthoma cells are lipid-laden macrophages because they contain lipid and immunohistochemically express CD68 and demonstrate neither mucin nor pigments [6, 7]. Endoscopically, xanthomas usually present as flat lesions and whitish yellow in color. Gastrointestinal xanthoma with polyp is extremely rare [8]. Lin et al. [1] reported a case of gastric hyperplastic polyps that showed collections of xanthoma cells in the lamina propria. Gencosmanoglu et al. [4] also reported a case of gastric xanthelasma within a hyperplastic polyp.

 In studies of gastric polyps, we found that hyperplastic polyps were the most common; nearly 85%–91% of all polyps were hyperplastic polyps [2, 3, 9]. In another report, the incidence of gastric hyperplastic polyps was reported to be 28.3% in one series of 5515 gastric polyps by Stolte et al. [10]. As gastric hyperplastic polyps are common, we should identify the relationship between gastric hyperplastic polyps and xanthoma. The coexistence of gastric hyperplastic polyp and xanthoma is rare, and its etiology remains obscure. It was speculated that the coexistence of these two lesions was likely to be related to associated gastric diseases such as chronic gastritis [1, 4]. On the other hand, there has been a report on association of gastric cancer (0–II a type) and xanthoma [11]. Muraoka et al. [11] speculated that gastric cancer could coincidentally arise close to a pre-existing xanthoma and recommended to perform a biopsy if gastric xanthoma associated with irregular protruding lesion. Thus we should make correct diagnosis whether the lesion is malignant or not if gastric xanthoma associates with protruding lesion. Endoscopists should pay particular attention to gastric malignant protruding lesions associated with gastric xanthoma. Magnification endoscopy may be useful for the detection of malignant lesion occurring in the gastric protruding lesion [12] because we had reported a case of gastric papillary adenocarcinoma occurring in a gastric hyperplastic polyp that can be diagnosed by magnification endoscopy [13]. If disappearance of the mucosal microstructure or irregular branched capillary is observed in the protruding lesion associated with gastric xanthoma by magnification narrow-band imaging endoscopy, the lesion has a possibility of malignancy and should be removed [13]. There was no evidence of malignancy in endoscopic findings (including magnification endoscopy) and histological findings in the present case.

 In conclusion, we reported an extremely rare case of gastric hyperplastic polyp with proliferation of xanthoma cells. In the present case, there was no evidence of malignancy. There are no reports describing the magnifying endoscopy (with narrow-band imaging) findings of the gastric hyperplastic polyp with proliferation of xanthoma cells. It is necessary to know that a gastric hyperplastic polyp may associate with gastric xanthoma, although such association is very rare.
